# Revising the Landscape of Cytokine-Induced Killer Cell Therapy in Lung Cancer: Focus on Immune Checkpoint Inhibitors

**DOI:** 10.3390/ijms24065626

**Published:** 2023-03-15

**Authors:** Rohulla Vaseq, Amit Sharma, Yutao Li, Ingo G. H. Schmidt-Wolf

**Affiliations:** 1 Department of Integrated Oncology, CIO Bonn, University Hospital Bonn, Venusberg-Campus 1, 53127 Bonn, Germany; rohulla.vaseq@ukbonn.de (R.V.); amit.sharma@ukbonn.de (A.S.); yutao.li@ukbonn.de (Y.L.); 2 Department of Neurosurgery, University Hospital Bonn, 53127 Bonn, Germany

**Keywords:** lung cancer, NSCLC, SCLC, CIK cells, immune checkpoint, PD-1, PD-L1, CTLA-4

## Abstract

Undeniably, immunotherapy has markedly improved the survival rate of cancer patients. The scenario is no different in lung cancer, where multiple treatment options are now available and the inclusion of immunotherapy yields better clinical benefits than previously used chemotherapeutic strategies. Of interest, cytokine-induced killer (CIK) cell immunotherapy has also taken a central role in clinical trials for the treatment of lung cancer. Herein, we describe the relative success of CIK cell therapy (alone and combined with dendritic cells as DC/CIKs) in lung cancer clinical trials and discuss its combination with known immune checkpoint inhibitors (anti-CTLA-4 and anti-PD-1/PD-L1). Additionally, we provide insights into the findings of several preclinical in vitro/in vivo studies linked to lung cancer. In our opinion, CIK cell therapy, which recently completed 30 years and has been approved in many countries, including Germany, offers tremendous potential for lung cancer. Foremost, when it is optimized on a patient-by-patient basis with special attention to the patient-specific genomic signature.

## 1. Introduction

**Figure 1:**
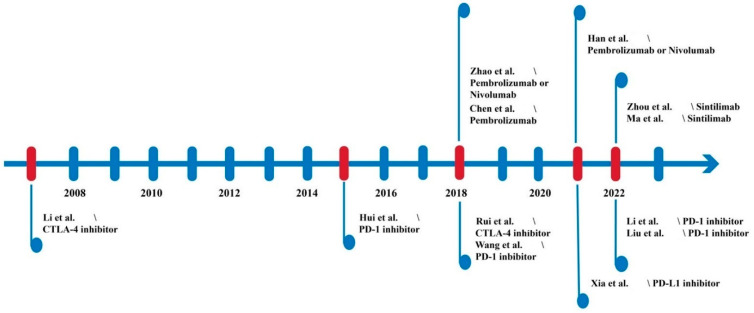
Timeline of CIK cell application with immune checkpoint inhibitors in the lung cancer context adapted from references [10,11,12,13,14,15,16,17,18,19,20,21].

Certainly, cancer is not limited to the dominance of a few cellular/molecular factors; rather, a large pool of cancer-causing lesions may emerge from the clinical and pathological stages. Owing to the extensive genomic sequencing approaches, both mutation-related and pathologically variable molecular features of lung cancers have been elaborated. In fact, an accumulation of such datasets has also helped to successfully and effectively define (diagnostically) diverse lung cancer types ranging from non-small cell lung cancer (NSCLC: squamous cell carcinoma and non-squamous cell carcinoma) to small cell lung cancer (SCLC: limited stage SCLC and extensive stage SCLC) [1,2,3,4]. However, it is still unclear how lung cancer cells can escape the immune system, posing a serious challenge to treatment resistance. Cancer, being a heterogeneous entity, grows abnormally with metastatic properties, and lung cancer, having an annual mortality rate of more than 80%, is no different in this regard. Certainly, immunotherapy has emerged as an innovative therapy for various cancers, and the growing success of immune checkpoint inhibitors (ICIs), the most representative immunotherapy, has led to effective treatment of cancer due to their durable anti-tumor effects. Among the checkpoint-blocking strategies, blockades for programmed cell death 1 (PD-1), programmed cell death ligand 1 (PD-L1), and cytotoxic T-lymphocyte associated protein 4 (CTLA-4) have gained increasing attention. These inhibitors, alone or in combination, improved treatment response and prolonged the survival time of NSCLC patients, which show superior efficacy to chemotherapy [2,5,6,7,8]. However, in a subset of patients, treatment resistance remains a major challenge. To overcome this, the combination of ICI with other therapeutic approaches such as adoptive cell therapy (ACT) is being pursued. From this perspective, cytokine-induced killer (CIK) cells have emerged as a feasible and effective prime candidate for adoptive immunotherapy [8,9]. Of interest, the synergistic effects of ICIs and CIK cells to increase the anti-tumor potency have been tested in several preclinical and clinical studies (Figure 1). With a special focus on lung cancer, we present here the current details and results of all preclinical and clinical studies performed using CIK cells in combination with ICIs.

## 2. CIK Cells as a Realistic Option in Cancer Immunotherapy

Recently, we have celebrated 30 years of CIK cell therapy [22]. Meanwhile, CIK cell treatment is licensed in many countries, including Germany. CIK cells as adoptive cellular immunotherapy were first described as an effective anti-tumor weapon in 1991 [23]. As the name implies, CIK cells are peripheral blood mononuclear cells (PBMCs) engineered by antibody/cytokine cocktails and expanded ex vivo for approximately 14 days. As a heterogeneous population of cells bearing the CD3+ and CD56+ labels, they contain a mix of CD3+CD8+, CD3+CD56+, and CD3+CD56- cells, whose function is primarily determined by their ability to function independently of major histocompatibility complexes (MHCs) and by their Natural Killer Group 2D (NKG2D) receptor activity. CIK cells have been demonstrated, by pre-clinical models and clinical trials, to be able to reveal a dominant anti-tumor activity in solid and non-solid tumors [24]. In addition to simple preparation methods, as a result of the treatment of CIK cells, various benefits are evident, including the prevention of recurrence, the improvement of quality of life, the improvement of progression-free survival and overall survival, the safety and tolerability throughout the treatment, and significant cytotoxicity against numerous types of cancer. To date, more than 80 clinical trials involving CIK cells have been performed [25]. One of the most important reasons for using CIK cells in clinics is the possibility of obtaining them also from PBMCs of healthy donors, especially when the health status of patients (the elderly or patients with immunodeficiencies) does not make this possible. However, studies indicate tumors can still progress even under strong immune pressure. It seems tumor microenvironment conditions composed of variants have an undeniable effect on immunotherapy strategies such as CIK cells [7]. It is, therefore, undeniable that designing a rational strategy that has a better outcome when it comes to fighting cancer is of vital importance. Numerous studies have indicated that combining CIK with other immunotherapy strategies greatly enhances the effectiveness of both treatments [25,26,27,28]. Since immune checkpoint targeting showed a considerable result in lung cancer, in this review, we want to evaluate the pre-clinical and clinical output of a combination of CIK cells and immune checkpoints in lung cancer.

## 3. CIK Cells Combined with PD-1/PD-L1 in Lung Cancer

One of the first clinical evidences of advanced squamous non-small cell lung cancer (NSCLC) with severe thrombocytopenia showed dramatic improvement after first-line treatment with pembrolizumab (anti-PD-1 monoclonal antibody (mAb)) and autologous CIK cells [10]. Considering the different role of PD-1 inhibitors in NSCLC patients with varying clinical and molecular features, this rare case report represents an important update demonstrating that therapy with a PD-1-blocking antibody and autologous CIK cells is well tolerated. Assuming that CIK cells might be partly exhausted before clinical transfusion, Zhang et al. suggested implementing combined treatment on CIK cells before transfusion via antibodies targeting PD-L1, lymphocyte activation gene 3 (LAG-3), T-cell immunoglobulin mucin-3 (TIM-3), and carcinoembryonic antigen-related cell adhesion molecule 1 (CEACAM-1) in order to improve the efficiency of CIK therapy for NSCLC patients [29]. In another study, PD-1 blockade in combination with CIK cells also showed promising clinical responses in two patients with metastatic renal cell carcinoma and NSCLC [11]. Liu et al. suggested that CD4+ T-cells are required to effectively reverse the functional exhaustion of CIK cells infiltration into NSCLC and restore the cytotoxicity of CIK cells through the IL-17/AKT/T-bet axis [9]. Recently, Zhou et al. conducted a phase IB study of autologous CIK cells in combination with Sintilimab (a monoclonal antibody against PD-1) plus chemotherapy in patients with advanced NSCLC [30].The authors clearly demonstrated well-tolerated effects and encouraging efficacy in patients with previously untreated advanced NSCLC. Ma et al. also recently evaluated the clinical efficacy of CIK cells combined with cytotoxic chemotherapy, followed by sintilimab maintenance, in ES-SCLC patients [12]. To investigate the potential impact of genetic background on the efficiency of CIK cells, Li et al. cultured three genetically distinct NSCLC cell lines with distinct rearrangements (EML4-ALK, KRAS mutation, and ROS1 rearrangement) in combination with PD-1 and an anaplastic lymphoma kinase (ALK) inhibitor [13]. The authors hypothesize that CIK therapy may be a potential alternative in NSCLC patients harboring EML4-ALK rearrangement. Likewise, a study performed whole-exome sequencing (WES) on samples from NSCLC patients treated with CIK cells and concluded that somatic copy number changes can predict clinical benefit in patients with lung adenocarcinoma treated with CIK cells plus chemotherapy [31].

Wang et al. also performed WES and transcriptomic analyses of tumor tissues and paired adjacent benign tissues collected from different subtypes of patients with NSCLC before CIK immunotherapy [32]. The authors concluded that CIK immunotherapy is more effective in patients with lung squamous cell carcinoma (SCC) than in lung adenocarcinoma. Han et al. recently conducted a retrospective study of PD-1-blocking antibodies (pembrolizumab or nivolumab) plus autologous CIK cells to evaluate the safety, efficacy, and impact of this treatment on immune function in patients with advanced NSCLC [14]. The authors concluded that the combination of CIK cells and a PD-1-blocking antibody appears to be well tolerated and has promising clinical activity, including high response rates and the potential for deep and durable responses. Some NSCLC subsets have been shown to express PDL-1, but those with positive PDL-1 and higher numbers of CD8+ tumor-infiltrating lymphocytes (TILs)—conventionally referred to as “hot tumors”—had a better objective response rate (ORR) with combination therapy of PD-1 inhibitors, CIK cells, and chemotherapy [30]. Studies indicate that the use of anti-PD-1 increases both NKG2D and CD56-positive cell populations [14,33,34]. In addition, Chen et al. [35] indicated that prolonged PD-1 blockade increased the CD3+CD56+ subpopulation rate with a higher level of NKG2D expression. Further, they demonstrated that the H1975 lung cancer cell line exhibited higher PDL-1 expression levels compared to other lung cancer cell lines and that CD3+CD56+ PD-1-blocked cells significantly improved their cytotoxicity with an increasing effector to target (E:T) ratio. Furthermore, the number of CD107a-positive CIK cells in the PD-1-blocked CIK cells was 1.5-fold higher than in CIK cells cultured with the tumor cell line alone. In vivo assays show that CIK cells inhibited with a PD-1 inhibitor are better at preventing tumor growth. Zhang et al. [36] indicated that stimulating CIK cells with Pseudomonas aeruginosa-mannose-sensitive hemagglutinin (PA-MSHA) can reduce the PD-1- and TIM-3-positive cell population as an adjuvant. Xia et al. showed that LINC01140 knockdown, along with CIK administration, suppressed the growth of subcutaneous lung cancer xenografts by decreasing PD-L1 expression in severe combined immunodeficient mice [15]. To mention, an interesting study describes that anti-PD-1 mAbs should be administered before injection of CIK cells to maximize the efficacy of therapy. Since administration after the injection of CIK cells significantly impairs the binding rate of anti-PD-1 mAbs to the PD-1 receptor on CIK cells [16]. Recently, a novel PD-1-blocking nanobody (PD-1 Nb20) in combination with a tumor-specific dendritic cell (DC)/tumor fusion cell (FC) vaccine was found to effectively enhance the in vitro cytotoxicity of CD8+ T-cells to kill cancer cells, including NSCLCs [32].

**Figure 2:**
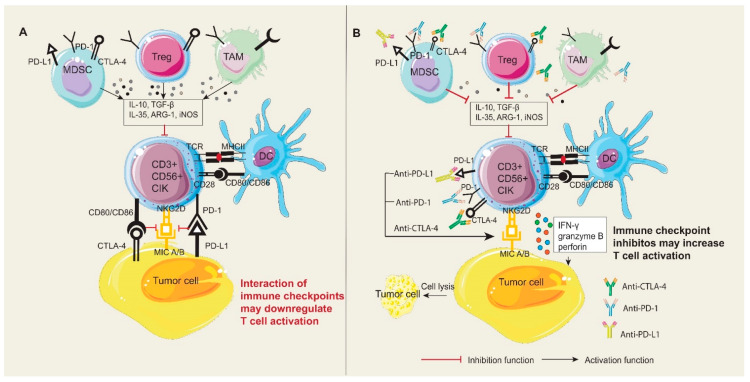
The potential effect of PD-1/PD-L1 interaction on CIK cells and tumor cells in the tumor microenvironment. (**A**) The interaction of immune checkpoints may downregulate T-cell activation. Upregulation of PD-1/PD-L1 or CTLA-4 on MDSCs, Tregs, and TAMs might increase the production of suppressor cytokines to restrain the function of CIK cells. DC cells capture the antigen with major histocompatibility complex (MHC) molecules, which bind to the TCR on T-cells to activate T-cells. However, the interaction of PD-1/PD-L1 or CD80/CD80 with CTLA-4 may damper the recognition of NKG2D on CIK cells with MICA/B on tumor cells and further influence the cytotoxicity of CIK cells against tumor cells. (**B**) Immune checkpoint inhibitors might amplify the cytotoxic potency of CIK cells through increasing NKG2D expression, inducing a higher CD3+CD56+ population, and reducing suppressor cytokines that are secreted by MDSCs, Tregs, and TAMs. DCs: Dendritic cells; MDSCs: Myeloid-derived suppressor cells; Tregs: Regulatory T-Cells; TAMs: Tumor-associated macrophages.

As compared to PDL-1, PDL-2 appears to have a less significant role in tumors and limited expression in dendritic cells, macrophages, and mast cells [37,38]. In addition, PDL-2 levels are not as high as PDL-1 levels in cancers, and in some cancers such as NSCLC, prostate, and endometrium, they have been reported [39,40,41]. In CIK cells, PD-1 expression is elevated in the early phase of generation but decreases in subsequent days; however, PDL-1 expression has been reported to be high in contrast to PDL-2, such that more than 50% of CIK cells are PDL-1 positive after 14 days, and similarly, PD-1 can induce quiescence in CIK cells after binding to CD80 [29]. The efficiency of anti-PDL-1 does not appear to be inferior to that of anti-PD-1. Anti-PD-1 antibodies were designed not to interfere with immune cell survival and function [42]. In extensive stage small cell lung cancer (ES-SCLC), atezolizumab, durvalumab, and pembrolizumab in combination with chemotherapy have been associated with better progression-free survival (PFS) and overall survival (OS) [1]. The complete details of the clinical trials and preclinical studies of CIK cells and PD-1/PD-L1 inhibitors are summarized in Table 1 and Table 2 and schematically shown in Figure 2.

## 4. CIK Cells Combined with CTLA-4 in Lung Cancer

CTLA-4 has been shown to be more highly expressed in tumor cells and correlates with poor prognosis [47,48]. However, Zhang et al. reported that CTLA-4 is not expressed in all NSCLC cell lines [49]. Of interest, two human mAbs that target CTLA-4, ipilimumab and tremelimumab, have already entered clinical trials. In ES-SCLC, the combination of ipilimumab and chemotherapy showed a better, although not significant, outcome [1]. Lung cancer patients receiving approved anti-CTLA-4 and anti-PD-1 antibodies achieved considerable results, though not to the extent of melanoma patients [50,51]. Hellman et al. suggest that the combination of nivolumab and ipilimumab is more likely to prevent NSCLC tumor growth compared with chemotherapy [52]. However, Jure-Kunkel et al. showed that the anti-CTLA-4 antibody can affect the lung cancer cell line M109 only in combination with ixabepilone [53]. Recently, a study provided a rationale for the benefit of aPD-1/aCTLA-4 combination therapy in malignant pleural mesothelioma (MPM) by demonstrating differences in the peripheral blood T-cell compartment in two phase II clinical trials evaluating aPD-1 monotherapy and aPD-1/aCTLA4 combination therapy [54]. The authors concluded that combined treatment with aPD-1 and aCTLA-4 triggered robust T-cell proliferation and activation in MPM patients, whereas aPD-1 monotherapy did not.

Although not enough data are available on the synergistic combination therapy of CIK cells with CTLA-4 in lung cancers, especially in the clinic, which may indicate an intriguing avenue about the need for further investigation, some studies suggest that the quality and quantity of CIK/DC-CIK cells against various tumor cell lines, in vivo and in vitro, are in some way intensified by inhibition of CTLA-4 (Table 2 and Figure 1). Interestingly, Rui et al. reported that CTLA-4 expression increases when PBMCs are induced into CIK cells during in vitro culture, and inhibiting CTLA-4 expression by shCTLA-4 lentiviral particles can enhance CIK cells proliferation ex vivo and their cytotoxicity toward A549 lung cancer cell lines [20]. A higher expression of CTLA-4 during CIK cell generation can be partially explained by the culturing conditions, which require IL-2 stimulation. In this context, Stojanovic et al. reported that CTLA-4 expression, in addition to CD-28, can be induced by IL-2 in natural killer (NK) cells from C57BL mice. Furthermore, IFN-γ secretion, one of the most important cytokines against tumor cells, can also be affected by higher CTLA-4 expression [45]. Conversely, Zhang et al. reported that CTLA-4 expression is high in the early stages of CIK cell transformation but slowly decreases in the following days [29]. Yuan et al. indicated that use of anti-PD1 and anti-CTLA-4 antibodies not only improved the expansion and differentiation of DC-CIK cells but also increased their cytotoxicity effects in a renal cancer cell line [55]. As aforementioned, the synergy of CIK cells with CTLA-4 in lung cancer clinical trials is still an open area that requires attention.

## 5. CIK Cells and Rational to Investigate Other Immune Checkpoint Inhibitors

The mature CIK cells (at day 15) have been shown to have high expression of PD-L1, LAG-3, TIM-3, and CEACAM-1 and low expression of the T-cell immunoreceptor with Ig and ITIM domains (TIGIT), the B- and T-lymphocyte attenuator (BTLA), PD-1, and CTLA-4 compared with their initial expression [29]. Therefore, it is reasonable to combine CIK cells with other ICIs as well. In this context, blocking the TIM-3 and PD-1 signaling pathways of DC-CIK cells with antibodies showed enhanced killing ability of DC-CIK cells in human lung adenocarcinoma A549 cells [21]. Poh et al. investigated whether blockade of inhibitory receptors on CIK cells by ICIs could enhance the antitumor efficacy of ICIs against hematologic malignancies [46]. The authors demonstrated that blockade of killer-cell immunoglobulin-like receptors (KIR), LAG-3, PD-1, and TIM-3, but not CTLA-4, resulted in a remarkable increase in the killing rate against defined targets.

## 6. DC/CIK Cells and Lung Cancer Clinical Spectrum

While only limited evidence is available from a handful of lung cancer trials, the results concerning DC/CIK cell therapy are encouraging. For instance, considering that the combination of dendritic cells (DCs) and CIKs can elicit an anti-tumor immune response, Zhang et al. investigated the feasibility of DCs/CIKs in combination with thoracic radiotherapy in patients with locally advanced or metastatic NSCLC, which confirmed the efficiency and safety of the treatment regimen [56]. Zhu et al. also suggested that combined DC-CIK therapy with synchronous radiotherapy and chemotherapy to treat stage IIIB NSCLC was superior to single synchronous radiotherapy and chemotherapy [57]. Chen et al. demonstrated that pembrolizumab (PD-1)-activated autologous DC-CIK cells exhibited a promising safety profile and showed an encouraging clinical response in patients with advanced solid tumors, including lung cancer [18]. Li et al. also evaluated the clinical efficacy of DC-activated CIK cell treatment following regular chemotherapy and the effects of this therapy on immune responses in patients with NSCLC after surgery [58]. Zhao et al. also suggested that a combined regimen of DC vaccination and CIK cell therapy with other treatments to overcome the systemic T helper 2 (Th2)-dominant immune response could improve the current clinical benefit [59]. Song et al. found that increased cycles of DC/CIK cell immunotherapy contributed to the decrease in Treg frequency and cancer recurrence rate in patients with resected NSCLC [60]. Zhang et al. showed that DC-CIK can induce an immune response against NSCLC, improve quality of life, and prolong overall patient survival without adverse side effects [61].

## 7. Conclusions and Future Prospects

We raised the scarcity of knowledge about CIK cells in combination with anti-PD-L1 and anti-CTLA-4 in lung cancer. ICIs can indeed cause a range of long-term side effects as they impair T-cell tolerance, though most of them tend to be mild [62,63]. A treatment with systemic steroids also helps well in some severe cases [64,65,66]. Importantly, ICIs are still considered less toxic compared to chemotherapy. Since these active compounds (e.g., ICIs) are used in multiple cancers (mainly in a combinatorial way), we believe that better tracking of these long-term effects is utterly important. Given that the molecular pathways targeted by ICIs also have a small overlap with microbiome-induced dysregulation [67], it is equally important to distinguish clinical phenotypes in cancer patients that are unrelated to antitumor activity. Pseudoprogression is another disconcerting feature of ICI administration; though it is rare, it has been reported in a few patients with NSCLC treated with ICIs [68,69]. To our knowledge, there is no evidence of pseudoprogression in CIK cell trails involving ICIs. Thus, it is reasonable to suggest that the adverse effects of ICIs (if any) appear to be negligible when they are combined with CIK cells.

Undeniably, ICIs are also expensive compared to other anticancer drugs, and the scenario is no different for lung cancer treatment [70,71,72]. However, the prognostic significance of PD-L1 continues to play a central role [73]. As evidenced in advanced-stage NSCLC, where positive PD-L1 expression has been associated with more aggressive pathologic features and a poorer prognosis [74]. Importantly, ICIs are well tolerated and significantly reduce the risk of death from lung cancer. Therefore, early screening for validated biomarkers that could predict the prognosis for immunotherapy is crucial for cost-effectiveness. Eventually, in the near future, the integration of datasets from NGS technologies may help evaluate appropriate drugs (ICI/cancer drugs) and estimate the potential clinical outcomes for individual patients. Based on our own experience and the literature information on lung cancer, it is reasonable to propose that CIK cells (alone and also as DC/CIKs) should be preclinically assayed with PD-L1 and CTLA-4. Given that epigenetic inhibitors can be easily tuned with CIK cells, they can be used in a complementary experimental setup with these particular ICIs. Most importantly, the genetic background underlying the preclinical lung cancer models should be at the forefront before reaching any conclusions. Regarding SCLC treated with CIK cells and an immune checkpoint inhibitor, remarkable results are not yet available. 

## Figures and Tables

**Table 1:** Clinical studies regarding the combination of CIK cells and immune checkpoint inhibitors.

Ref.	Clinical Trial	Phase	Tumor Entity	Treatment Regime	Pre-Treated	LC Patients (n)	Patients with CIK Therapy (n)	Status (for Registered Clinical Trials), Study Start Date	Median PFS/Month (CIK vs. Control)	Median OS/Month (CIK vs. Control)	ORR %(CIK vs. Control)	DCR Rate%	Adverse Effects
[30]	NCT03987867	I	AS & nS NSCLC	Auto CIK + SIN + chemo	No	34	34	Results published	19.3 (all patients)	20.3 *	82.4	100	Neutropenia, fatigue, nausea, leukopenia, thrombocytopenia, pneumonia, cardiomyopathy, dysphagia, rash, and cough.
[12]	NCT03983759	II	ES-SCLC	(Chemo + Auto CIK) as first line + SIN as second line	No	13	13	Results published	5.5	11.8	76.9	100	First line: anemia, thrombocytopenia, nausea, vomiting, leukopenia, rash, anorexia, and fatigue. Second line: increased aminotransferase.
[14]	-	Retro	AS & nS NSCLC	Auto CIK + PEM or NIV	-	7	7	-	9.7 vs. 1.3	21.7 vs. 28.4	42.86 vs. 9.09	57.14 vs. 45.45	Combination= pneumonia and exfoliative dermatitis. PD-1 inhibitor alone= adrenal insufficiency and hypothyroidism.
[17]	-	Retro	Advanced NSCLC	R-CIK + PEM or NIV	DT	7	3	-	TTP = 4.8	Not reached	28.6	85.7	Fatigue, anorexia, leukopenia, fever, rash, and interstitial pneumonitis.
[18]	-	I	ST include NSCLC	PEM-activated autologous DC-CIK cells	-	3	3		5.4 ^†^	9 ^†^	0	33.3	Observed in 64.5% of patients ^†^: fever, chills, anemia, increased AST, increased ALT, decreased albumin, leukopenia, thrombocytopenia, vitiligo, and hypothyroidism.
-	NCT03190811	Pros, I, II	ST include LC	Auto DC/CIK + PEM	Yes, ‡	100	-	RNP, Sep 2016	-	-	-	-	-
-	NCT03282435	Pros, I	NSCLC	Auto CIK + Anti PD-1	-	30	-	RNP, Jan 2018	-	-	-	-	-
-	NCT04836728	Pros, II	NSCLC	SIN + Chemo ± CIK cells	No	156	-	RNP, Apr 2021	-	-	-	-	-
-	NCT02886897	Pros, I, II	SL include LC	Auto CIK + Anti PD-1	No	50	-	RNP, Jul 2016	-	-	-	-	-
-	NCT03360630	Pros, I, II	NSCLC	PEM ± Auto DC/CIK cells	Yes, ‡	60	-	RNP, Nov 2016	-	-	-	-	-
-	NCT03815630	Pros, Early I	ST	PEM + Auto DC/CIK cell	Yes, chemo	100	-	RNP, Feb 2019	-	-	-	-	-

* (in squamous patients), not reached in all patients; ^†^ In out of 31 enrolled patients; ^‡^ standard therapy or declined chemotherapy/radiotherapy. Abbrivations: Cryo: Cryotherapy; LC: Lung cancer; PEM: Pembrolizumab; DC: Dendritic cells; RNP: Results not published; chemo: chemotherapy; Pros: Prospective; ST: Solid tumor; SIN: Sintilimab; AST: Aspartate transaminase; ALT: Alanine transaminase; NIV: Nivolumab; Retro: Retrospective; AS: Advanced Squamous; nS: non-squamous; PFS: progression-free survival; OS: overall survival; ORR: Objective response rate; DCR: Disease Control Rate.

**Table 2:** Pre-clinical and case studies regarding CIK cells and immune checkpoint inhibitors.

Ref	Cell Setting	Immune Checkpoint	Type of Inhibitor/Modulator	Type of Cancer	Output
[16] (2022)	CIK cells	PD-1	Anti-PD-1 + pemetrexed	A549, H1299	High CIK cell efficacy in prior anti-PD-1 mAb-treated mice compared with treating mice after CIK cell infusion.
[13] (2022)	CIK cells	PD-1	NIV + crizotinib (ALK inhibitor)	NCI-H2228 (EML4-ALK) A549 (KRAS mutation) HCC-78 (ROS1 rearrangement)	Reduction of PD-1 surface expression after antibody usage; Higher IFN-γ secretion; Higher granzyme B in CD3+CD56+; Higher Fas-L expression on CIK cells; Lower non-specific cytotoxicity (an adverse effect) in combinational therapy.
[9] (2022)	CD4- CIK cells	PD-1	Anti PD-1	A549	Higher CD56+ CIK cell subpopulation; Lower PD-1+ TIM-3+ CIK cells; Higher IFN-γ+Gzm B+ cell population.
[43] (2021)	DC-CIK cells	CTLA-4 of CIK cells	Nb36 nanobody	Hepatocellular carcinoma	Increased CTLA-4 expression during CIK cell generation; Higher proliferation and differentiation by CTLA-4 inhibition; Higher pro-inflammatory cytokine secretion by CTLA-4 inhibition; Higher survival rate by decreasing tumor growth in tumorized mice.
[15] (2021)	CIK cells	PD-L1	LINC01140	Lung cancer	LINC01140 knockdown lung cancer treated with CIK cells in vivo compared with the control group: • Higher regression of tumor growth; • Higher IL-2, TNF-α, IFN-γ; • Higher CD3+CD56+ CIK cell population; In co-culturing of CIK and LINC01140 knockdown lung cancer cells compared with non-knockdown tumor cells: • Higher IFN-γ secretion; • Lowe tumor cell viability; • Lower apoptotic CIK cells; LINC01140 expression inhibits PDL-1 mRNA inhibition, which leads to tumor cell invasion and immune evasion.
[44] (2020)	CIK cells	CTLA-4 of CIK cells and cell lines, PD-1	IPI	A-498 and Caki-2 renal cell lines	No differences in tumor cell viability by CTLA-4 inhibition; Higher CIK cell proliferation by CTLA-4 inhibition; No differences in CIK cell cytotoxicity by CTLA-4 inhibition; Higher IFN-γ secretion by CTLA-4 inhibition.
[45] (2019)	DC-CIK cells	CTLA-4 of CIK cells, PD-1	Anti CTLA-4 antibody	786 and ACHN (renal cell cancer)	Anti-CTLA-4 acts weaker than anti-PD1; Higher proliferation and differentiation by CTLA-4 inhibition; Higher cytotoxicity by CTLA-4 inhibition; Higher CD62L^low^CD44^hi^ cells by CTLA-4 inhibition; Higher pro-inflammatory cytokine secretion (IFN-γ and TNF-α) and lower suppressor cytokines (IL-10) by CTLA-4 inhibition.
[11] (2018)	Auto CIK cells	PD-1	PEM	Squamous NSCLC (Case report)	After 185 days of treatment, the patient is still in remission; No adverse effect has been seen.
[20] (2018)		CTLA-4 of CIK cells	shCTLA-4 lentiviral particles	A549	Increased CTLA-4 expression during CIK cell generation; Higher proliferation by CTLA-4 inhibition; Higher proliferation by CTLA-4 inhibition.
[34] (2016)	CIK cells	PD-1, PDL-1	Anti PD-1, Anti PDL-1	Gastric cancer and colorectal cancer cell line	Higher PD-1/PDL-1 expression on CIK cells following co-culture with the MGC803 cell line; Higher CIK cell cytotoxicity toward tumor cells after combining with either anti-PD-1 or anti-PDL-1 in vivo and in vitro; Higher IFN-γ, CD107a, and NKG2D expression are accompanied by impaired CTLA-4 and LAG-3 signaling by inhibiting PD-1/PDL-1 binding on CIK cells.
[46] (2016)	CIK cells	CTLA-4, KIR, LAG-3, PD-1, TIM-3	IPI	ALL, AML, MM, and U937, Raji	No differences in CTLA-4 expression during CIK cell generation; Expression of CTLA-4 ligands on tumor cells; No differences in CIK cell cytotoxicity by CTLA-4 inhibition compared to KIR, LAG-3, PD-1, and TIM-3 blockade; No autologous reaction to blocking CTLA-4 CIK cells.

Abbreviations: AS: Advanced Squamous; PEM: Pembrolizumab; IPI: Ipilimumab; NIV: Nivolumab.

## Data Availability

Not applicable.
